# Human Subcutaneous Dirofilariasis, Russia

**DOI:** 10.3201/eid1301.060920

**Published:** 2007-01

**Authors:** Laura H. Kramer, Vladimir V. Kartashev, Giulio Grandi, Rodrigo Morchón, Sergei A. Nagornii, Panagiotis Karanis, Fernando Simón

**Affiliations:** * Università degli Studi di Parma, Parma, Italy; † Rostov State Medical University, Rostov-on-Don, Russia; ‡ Universidad de Salamanca, Salamanca, Spain; § Rostov Research Institute of Microbiology and Parasitology, Rostov-on-Don, Russia; ¶ University of Cologne, Cologne, Germany

**Keywords:** Human subcutaneous dirofilariasis, Dirofilaria repens, Russia, histology, serology, PCR, dispatch

## Abstract

We report 14 cases of human subcutaneous dirofilariasis caused by *Dirofilaria repens*, diagnosed from February 2003 through July 2004, in patients from Rostov-on-Don, Russia. Serologic analysis showed evidence of high risk of exposure to *D. repens*. Surveillance studies on prevalence and prevention effectiveness of canine infection are needed to control this emerging zoonosis.

Human subcutaneous dirofilariasis (HSD) is a zoonotic filariasis caused by infection with several species of worms belonging to the genus *Dirofilaria*; most documented cases are attributed to *Dirofilaria repens* (*1*). Dirofilarias are natural parasites of a great variety of animals and, with the exception of *D*. *immitis*, live in the subcutaneous tissue of their hosts, produce circulating microfilariae, and are transmitted by mosquitoes (*2*). The principal reservoir of *D*. *repens* is the dog. Humans are accidental hosts with patent infections being extremely rare. Differential diagnoses of HSD include neoplasia and other granulomatous diseases, and a definitive diagnosis usually requires surgical removal and examination of a granuloma.

Current epidemiologic studies indicate that human dirofilariasis is increasing in prevalence, and several authors have recently described it as an emerging disease in different areas of the world. Pampiglioni et al. (*3*) reported 60 new cases in Italy. Eleven cases of subcutaneous dirofilariasis have been diagnosed near Moscow, Russia (*4*). Cases have been reported in Taiwan (*5*). A total of 48% of human dirofilaosis cases reported in France have been diagnosed in the previous 10 years (*6*). We report 14 confirmed cases of HSD diagnosed from February 2003 through July 2004 in patients from Rostov-on-Don in southeastern Russia, and serologic evidence of high risk of exposure to *D*. *repens* infection in the local population.

## The Study

Skin nodules were removed from 14 patients from February 2003 through July 2004. Eleven patients were female, and 3 were male (age range 23–66 years). Nodule localization included the head, trunk, inguinal area, and feet (Table). Nodules ranged from ≈4 mm to ≈2 cm and were examined by routine histologic analysis. In 1 case, genomic DNA was extracted from an intact worm excised from a nodule by using the NucleoSpin Tissue procedure (Macherey-Nagel, Düren, Germany). PCR was conducted according to the procedure of Favia et al. (*7*). Amplicons were visualized under a UV transilluminator after electrophoresis on 1.5% agarose gels and staining with ethidium bromide (0.5 μg/mL). Gels were scanned by using a digital photograph system (Gel Logic 100, Eastman Kodak, Rochester, NY, USA).

**Table:** Sex, age, and location of nodules of 14 patients with subcutaneous dirofilariasis diagnosed in Rostov-on-Don, Russia, February 2003–July 2004

Patient	Sex	Age, y	Location of nodule
1	F	29	Temporal area
2	M	40	Left upper eyelid
3	F	61	Left groin
4	F	54	Left shoulder
5	F	43	Left shoulder
6	F	23	Oral cavity
7	M	38	Forehead
8	F	39	Left shoulder
9	F	43	Back
10	F	66	Left breast
11	F	43	Cheek
12	F	28	Temporal area
13	M	55	Left foot
14	F	35	Right foot

Serum samples were taken at the time of nodule excision surgery for 9 of the 14 patients. These samples were analyzed with an ELISA for antibody response to *D*. *repens* somatic antigen, as described (*8*). Briefly, 96-well microplates were coated with 0.6 μg of *D*. *repens* somatic antigen prepared according to the procedure of Prieto et al. (*9*). All serum samples were analyzed at a dilution of 1:30, and anti-human peroxidase-conjugated immunoglobulin G was used at a dilution of 1:4,000. Optical densities (ODs) were measured at 492 nm in an Easy Reader (Bio-Rad Laboratories, Hercules, CA, USA). Positive antibody response was defined as an OD value greater than the mean value ± 3 standard deviations for 14 serum samples from clinically healthy blood donors living in a *D*. *repens*–free area. A total of 317 serum samples from a random hospital population in Rostov were divided into categories on the basis of sex and age and analyzed by ELISA as described above.

**Figure 1:**
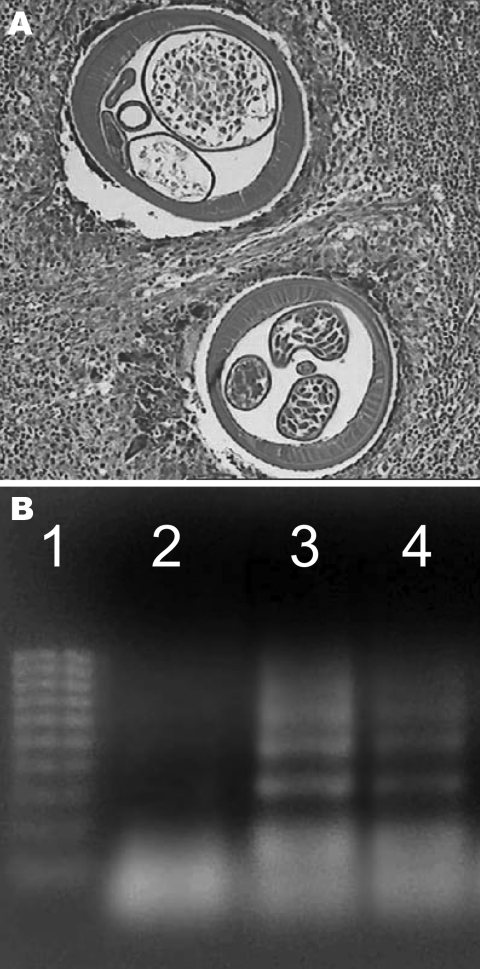
A) Histologic analysis of skin nodules caused by human subcutaneous dirofilariasis. Cross-sections of *Dirofilaria repens* surrounded by an inflammatory granuloma. Note the uteri with developing embryos (hematoxylin and eosin stain, original magnification 10×). B) Analysis of patient samples by agarose gel electrophoresis. Lane 1, 100-bp DNA molecular mass weight marker; lane 2, negative control; lane 3, positive control; lane 4, patient sample showing banding pattern typical of the positive control and similar to the banding pattern described in the original protocol (*7*).

**Figure 2:**
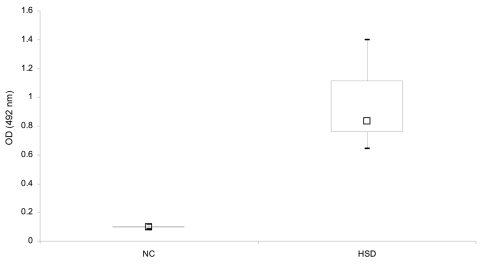
Serologic analysis (box and whisker plot) for antibodies to *Dirofilaria repens* somatic antigen from normal controls (NC) and patients with human subcutaneous dirofilariasis (HSD). The horizontal line shows the optical density (OD) values of 25–75% of the examined sera. The large box shows OD values between the first and third quartiles, the small box shows the median, and error bars show maximum and minimum OD values. A positive antibody response was defined as an OD value greater than the mean value ±3 standard deviations from 14 normal controls.

Routine histologic analysis of all nodules showed an intense inflammatory granuloma around several cross-sections of filarial nematodes (Figure 1A). The average diameter of adult worms was ≈450 μm. These worms had 95 longitudinal ridges on the external cuticle, 2–5 chord nuclei per section, and robust muscle cells, all of which are characteristic features of *D*. *repens* (*10*). Results of PCR analysis of a worm excised from 1 of the nodules was specific for *D*. *repens* (Figure 1B). All 9 patients with HSD had significantly higher OD values for total serum immunoglobulin G against *D*. *repens* somatic antigen (p = 0.001) than controls (Figure 2). Thirty-three (10.4%) serum samples from a random hospital population from the area had positive OD values for antibodies against *D*. *repens* somatic antigen. Prevalence of infection was higher in males (28/235, 12%) than in females (5/81, 6%) and in persons >60 years of age (25%) (data not shown).

## Conclusions

The cases of HSD described were all diagnosed in patients who had never traveled outside the Rostov area. This is the highest number of cases of HSD diagnosed worldwide in such a short period. Histologic analysis and PCR indicate that *D*. *repens* is the causative agent of HSD in this area, and serologic analysis suggests that the risk for exposure is high.

Domestic and wild canids are definitive hosts of *D*. *repens*; the dog is the principal reservoir. No epidemiologic data are available on infection prevalence in dogs in southern Russia. In Piedmont, Italy, a region with a high incidence of human dirofilariasis, a survey of dogs conducted in 1966–1967 and repeated in 1991–1992 (*11*) showed a marked increase in the number of infected animals and size of the endemic area. Any increase in the population of vectors and infection of the reservoir may likely be associated with an increase in human dirofilariasis.

Information is also lacking on which mosquito vectors are involved in transmission of *D*. *repens* in the study area. In other geographic areas where human dirofilariasis is endemic, changes in climatic conditions (temperature, relative humidity, rainfall, rate of evaporation) favor the development of vector mosquitoes (*12*) and of the larval phase of the nematode in the vector.

Medical awareness of infection risk is essential for a correct diagnosis, and the use of serologic analysis for *D*. *repens* somatic antigen merits further study as a diagnostic aid. Further monitoring of the HSD situation in this area is needed to establish guidelines for preventive measures, including effective chemoprophylaxis in animals.
